# Sucrose accumulation in sweet sorghum stems occurs by apoplasmic phloem unloading and does not involve differential *Sucrose transporter* expression

**DOI:** 10.1186/s12870-015-0572-8

**Published:** 2015-07-30

**Authors:** Saadia Bihmidine, R. Frank Baker, Cassandra Hoffner, David M. Braun

**Affiliations:** Division of Biological Sciences, Interdisciplinary Plant Group, and Missouri Maize Center, University of Missouri, 110 Tucker Hall, Columbia, MO 65211 USA; University of Missouri Molecular Cytology Core, 120 Bond Life Sciences Center, 1201 Rollins Street, Columbia, MO 65211-7310 USA; Sigma-Aldrich Biotech, 545 S. Ewing, Saint Louis, MO 63103 USA

**Keywords:** Apoplasm, Carbohydrate partitioning, Carboxyfluorescein, Parenchyma, Phloem, Sorghum, Stem, Sucrose, SUT, Symplasm

## Abstract

**Background:**

Sorghum (*Sorghum bicolor* L. Moench) cultivars store non-structural carbohydrates predominantly as either starch in seeds (grain sorghums) or sugars in stems (sweet sorghums). Previous research determined that sucrose accumulation in sweet sorghum stems was not correlated with the activities of enzymes functioning in sucrose metabolism, and that an apoplasmic transport step may be involved in stem sucrose accumulation. However, the sucrose unloading pathway from stem phloem to storage parenchyma cells remains unelucidated. Sucrose transporters (SUTs) transport sucrose across membranes, and have been proposed to function in sucrose partitioning differences between sweet and grain sorghums. The purpose of this study was to characterize the key differences in carbohydrate accumulation between a sweet and a grain sorghum, to define the path sucrose may follow for accumulation in sorghum stems, and to determine the roles played by sorghum SUTs in stem sucrose accumulation.

**Results:**

Dye tracer studies to determine the sucrose transport route revealed that, for both the sweet sorghum cultivar Wray and grain sorghum cultivar Macia, the phloem in the stem veins was symplasmically isolated from surrounding cells, suggesting sucrose was apoplasmically unloaded. Once in the phloem apoplasm, a soluble tracer diffused from the vein to stem parenchyma cell walls, indicating the lignified mestome sheath encompassing the vein did not prevent apoplasmic flux outside of the vein. To characterize carbohydrate partitioning differences between Wray and Macia, we compared the growth, stem juice volume, solute contents, *SbSUTs* gene expression, and additional traits. Contrary to previous findings, we detected no significant differences in *SbSUTs* gene expression within stem tissues.

**Conclusions:**

Phloem sieve tubes within sweet and grain sorghum stems are symplasmically isolated from surrounding cells; hence, unloading from the phloem likely occurs apoplasmically, thereby defining the location of the previously postulated step for sucrose transport. Additionally, no changes in *SbSUTs* gene expression were detected in sweet vs. grain sorghum stems, suggesting alterations in *SbSUT* transcript levels do not account for the carbohydrate partitioning differences between cultivars. A model illustrating sucrose phloem unloading and movement to stem storage parenchyma, and highlighting roles for sucrose transport proteins in sorghum stems is discussed.

**Electronic supplementary material:**

The online version of this article (doi:10.1186/s12870-015-0572-8) contains supplementary material, which is available to authorized users.

## Background

The human population is projected to reach over nine billion people by 2050; hence, crop productivity for food and energy security must be substantially increased to provide for the expected demand [1–3]. Because little additional arable land will be available for expanded crop cultivation, these increases will need to be derived from improved crop performance. Some of the agricultural increases could be provided by better management practices, improved abiotic and biotic stress tolerance to prevent crop loss, and enhanced delivery of assimilates into storage organs to increase yield. However, for many crops, the pathways followed by photoassimilates from their sites of synthesis to their deposition in storage tissues are not well defined. Within this context, carbohydrates stored in the seeds of grasses provide the majority of humanity’s daily caloric intake. Additionally, renewable sources of energy derived from plant biomass are being developed by using soluble sugars stored in the stems of sweet sorghum (*Sorghum bicolor* L. Moench) and sugarcane (*Saccharum officinarum* L.), or those converted into lignocellulose in the stems of bioenergy sorghums, switchgrass (*Panicum virgatum* L.), and *Miscanthus x giganteus* [4–11]. Hence, strategies to improve nutrient delivery to harvested organs for food, feed, fiber, and fuel uses hinge upon the transport routes for photoassimilates, and the transporters involved in long-distance allocation [12–14].

Carbohydrate partitioning is the process by which photoassimilates are distributed throughout the plant from their sites of synthesis in leaves to their incorporation into storage products, such as in fruits, seeds, tubers, and stems [9, 15–22]. In most crop plants, sucrose is the soluble carbohydrate that is transported from photosynthetic leaves to non-photosynthetic tissues, which import this fixed carbon for utilization and storage. Tissues such as leaves that export fixed carbon are termed sources, whereas tissues that import and store carbohydrates are referred to as sinks. Transport of assimilates through the plant occurs in the phloem tissues of veins [23, 24]. The rate of phloem transport of assimilates can be controlled at either the source or sink tissues, depending upon the developmental stage of the plant and the environment [24, 25]. The differential capacity of distinct sink tissues to compete for the import and utilization of photoassimilates, also known as sink strength, can control phloem transport and allocation of carbohydrates [26–29].

Within the source tissues, the loading of sucrose into the phloem can involve either symplasmic or apoplasmic pathways [21, 30]. In symplasmic loaders, sucrose diffuses directly between cells and into the sieve element/companion cell complexes of the phloem through plasmodesmata, connections that link the cytoplasm between cells. In apoplasmic loaders, sucrose can move symplasmically between cell types, but is ultimately exported into the extracellular space (the apoplasm) of the phloem prior to subsequent uptake across the plasma membrane of the sieve element/companion cell complexes. With the possible exception of rice (*Oryza sativa L.*), which has been suggested may use symplasmic phloem loading [31, 32], but see [12], the path for sucrose entry into the phloem in the leaves of grasses such as sugarcane, maize (*Zea mays* L.), wheat (*Triticum aestivum* L.), and barley (*Hordeum vulgare* L.), is proposed to occur by apoplasmic phloem loading [33–37].

Apoplasmic phloem loading requires multiple classes of sucrose transport proteins for sucrose to traverse cell membranes. Sucrose transporters (SUTs) are H^+^/sucrose symporters that use the energy stored in the proton motive force to transport sucrose across a membrane. Phylogenetic analyses have divided the SUTs into multiple groups or types [38–42]. Different family members have been proposed to function on the plasma membrane to load sucrose into the phloem [15, 39], or on the tonoplast to transport sucrose from the vacuole into the cytoplasm [43–45]. SWEETs are another class of sugar transport proteins, and they have been proposed to function as uniporters that facilitate the movement of sugars down a concentration gradient, with clade III members transporting sucrose across membranes [46–50]. Tonoplast sugar transporters (TSTs, also called tonoplast monosaccharide transporters) are a third class of sucrose transport proteins, and they function as H^+^/sucrose antiporters to transport sucrose into the vacuole [51, 52]. SUTs, SWEETs, and TSTs are all thought to play important roles in carbohydrate partitioning and storage in food and fuel crops [9, 13, 15, 17, 26, 48, 50, 52, 53].

Once entered into the phloem, sucrose is transported long-distance to sink tissues via bulk flow [28, 54, 55]. Depending on the plant, tissue, and developmental stage, sucrose can exit the phloem either symplasmically or apoplasmically [12, 24, 54, 56, 57]. If sucrose follows a symplasmic route, it can move out from the phloem sieve tube through plasmodesmata into the adjacent cells. Sucrose accumulation within sugarcane stem internodes has been suggested to utilize a symplasmic phloem unloading pathway followed by post-phloem sucrose movement through plasmodesmata to storage within stem parenchyma cells [26, 58–60]. Alternatively, if an apoplasmic path is used, sucrose must be effluxed across the sieve tube plasma membrane prior to uptake from the apoplasm into adjacent cells, such as in the maize and sorghum grain [26, 61, 62].

Grain sorghum is an important staple crop in Africa and China that stores carbohydrates as starch in the seed [63–65]. Sweet sorghum is a different variety that has been selected to store large quantities of soluble sugars (mostly sucrose) in the stem, and has been advanced as a valuable feedstock for producing ethanol from plants [5, 9, 66]. Sweet and grain sorghums are genetically closely related and are both classified as *S. bicolor*. Population genetic analyses have found that sweet vs. grain sorghum distinctions, while useful for breeding and phenotypic classification, are not distinguishable along racial subtypes by molecular markers [67–70]. Nonetheless, the different terminal sink tissues and storage forms for carbohydrate deposition in grain vs. sweet sorghums makes them an ideal comparative system to study the genes and processes controlling carbohydrate partitioning in grasses [71–75].

Previous research investigating sucrose accumulation in sweet sorghum stems found that sucrose accumulation began at the start of the reproductive phase, and that the activities of sucrose metabolizing enzymes were not correlated with sucrose concentration [76]. Further studies determined that sucrose accumulation could begin pre-reproduction in some sweet sorghum cultivars, but again found no correlation with the activities of enzymes involved in sucrose metabolism [77]. Based on these data, the authors of both studies suggested that transport of sucrose into the stem parenchyma likely underpinned stem sucrose accumulation patterns. Consistent with the lack of correlation to the activities of sucrose catabolic enzymes, additional investigations suggested that sucrose could be taken up directly into mature sweet sorghum stem parenchyma cells without first being cleaved into hexoses and resynthesized [78, 79]. Using asymmetrically radiolabeled sucrose infused into mature sorghum stems, it was also reported that sucrose movement in mature internodes included an apoplasmic transport step [79], implicating the function of sucrose transport proteins.

The sorghum genome contains six *SUT* genes [39]. *SbSUT2* is a member of the group 4/type III clade and is predicted to be localized to the tonoplast. The other five sorghum *SUT*s are predicted to be localized to the plasma membrane and belong to groups 1, 3, or 5/type II. *SbSUT1* is orthologous to, and likely has a conserved function with, the maize *ZmSUT1* gene, which has been shown by expression, biochemical activity, and genetic analyses to function in sucrose phloem loading [80–84]. The functions of the other sorghum *SUT* genes remain unknown. From expression studies, *SbSUT*s are broadly expressed in both sink and sources tissues, with different family members showing distinct expression patterns [75, 85, 86]. In comparing *SbSUT* expression levels between grain and sweet sorghum tissues, differences have been reported for all genes, with the exception of *SbSUT3,* whose expression has not been detected. Whether these expression differences contribute to differences in carbohydrate partitioning between grain and sweet sorghum is unknown.

In this study, we used a combination of morphological, biochemical, photosynthetic, cell biological, and gene expression studies to understand the major differences between sweet vs. grain sorghum in regards to whole-plant carbohydrate partitioning, the transport path of sucrose in the stem, and the roles of *SbSUT*s in stem sucrose accumulation. To accomplish these aims, we compared a high biomass sweet sorghum cultivar, Wray, which produces a tall stem containing large quantities of soluble sugars as the principal stored form of carbohydrate, with a grain sorghum cultivar, Macia, which is shorter, but produces a large panicle with many seeds storing starch. The cultivar Wray was developed to have very high sugar content in the stem [68], whereas the cultivar Macia was developed for high grain yield and has been sequenced [87, 88]. These cultivars were selected for the current study because 1) they have been used in multiple other reports [68, 70, 89], and therefore have ample background information, and 2) they are highly divergent at the phenotypic level, and hence, might differ in the control of carbohydrate partitioning. In particular, we investigated *SbSUT* expression patterns to ascertain whether any of these genes might correlate with sugar accumulation in sweet sorghum stems. Based on our data, a model is proposed for the pathways for sucrose movement into sorghum stem storage parenchyma cells and the possible roles for different sucrose transport proteins.

## Results

### Whole-plant phenotype, biomass, and yield measurements

**Fig. 1:**
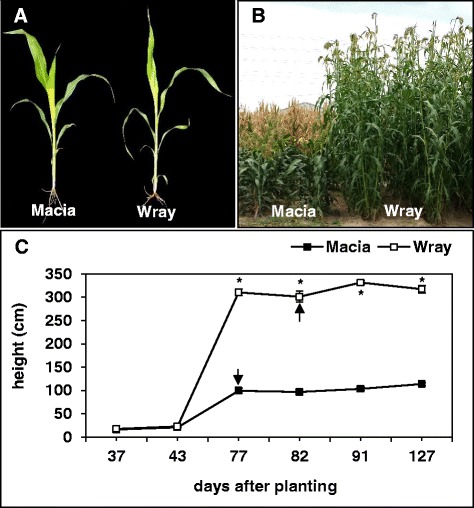
Comparison of the growth of grain (*cv.* Macia) and sweet (*cv.* Wray) sorghum plants. A side-by-side comparison of plants collected from the field at the early vegetative stage (**a**). Plants at maturity (**b**). A graph of the height of Macia and Wray plants measured in cm at different days after planting (**c**). Values are means ± SE of N = 10 plants, an asterisk indicates significantly different means between the two lines at *p* ≤ 0.05, and the arrows indicate the anthesis time for each line. Macia = black squares, and Wray = white squares

**Fig. 2:**
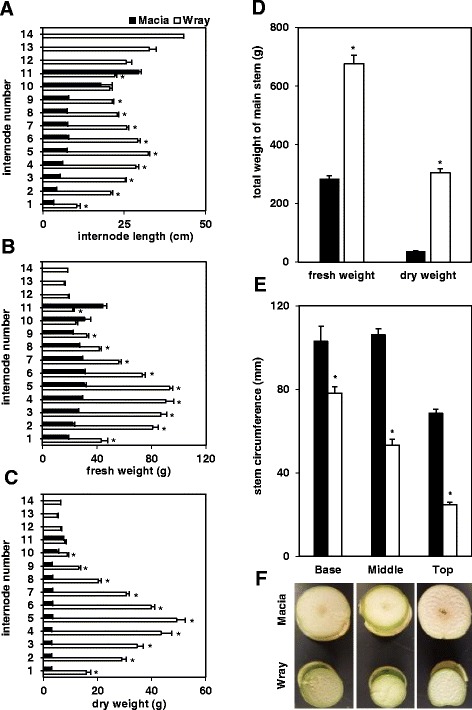
Differences in stem biomass parameters between Macia and Wray plants. Individual internode number and length in cm (**a**). Individual internodes fresh weight (**b**). Individual internodes dry weight (**c**). Total stem fresh and dry weight (**d**). Stem circumference measured in mm at different heights on the main stem: at the top internode, the mid-height internode, and the internode located at the base (**e**). Values are means ± SE, an asterisk indicates significantly different means between the two lines at *p* ≤ 0.05 on N = 10 (panels **a-d**) and N = 5 (panel **e**) plants, respectively. Macia = black bars, and Wray = white bars. Cross-sections of internodes taken at the same position where the circumference was measured (**f**)

To understand how and when Macia and Wray differ in terms of growth, yield, and carbohydrate allocation, we characterized plant growth, anthesis, biomass accumulation, and the total solutes in the stem juice, which is composed primarily of apoplasmic fluid, cytoplasm, and vacuolar sap, at multiple stages throughout their lifecycle. The early seedling growth of Macia and Wray appeared very similar (Fig. 1a). However, a number of morphological differences between the two cultivars emerged over time (Fig. 1b, c, and Additional file 1: Table S1). Beginning in the late vegetative stage (after 43 days after planting (DAP)), Wray developed taller stems as compared to Macia, with the difference in plant height increasing and being maintained throughout the season (Fig. 1b, c). In association with the increased stem height, Wray flowered an average of five days later than Macia (Fig. 1c). Additionally, Wray produced higher stem biomass compared to Macia (Fig. 2). Specifically, the total fresh and dry weight of the main stem collected at harvest was significantly higher in Wray than Macia (Fig. 2d). With the exception of the top one to two internodes, this difference was also reflected for each individual internode (Fig. 2b, c). Internode weights from Macia and Wray showed about a two-fold and nine-fold variation, respectively. Although Wray showed a significant increase in stem biomass, Macia displayed shorter but thicker stems (Fig. 2a, e, f). However, apart from the top two internodes, the significantly greater length of most of the internodes in Wray contributed more to the mass per internode than the greatly increased stem thickness in Macia (Fig. 2a-c, e, f). Therefore, Wray outperformed Macia at the level of biomass accumulation, as would be predicted for a sweet sorghum cultivar.

**Fig. 3:**
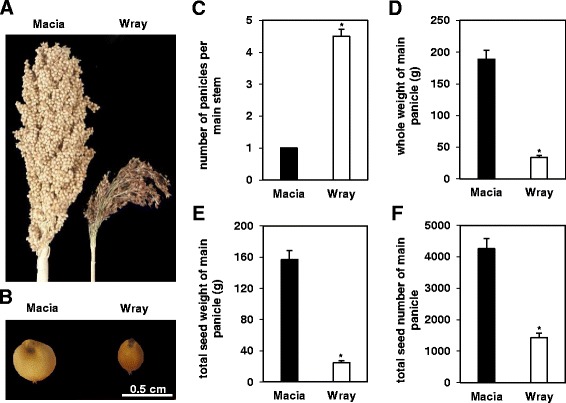
Differences in seed yield parameters between Macia and Wray plants. Images of side-by-side comparison of whole panicles (**a**) and single seeds (**b**). The graphs represent the number of panicles per main stem (**c**), whole panicle dry weight (**d**), total seed weight of the main panicle (**e**), and total seed number of the main panicle (**f**) of Macia and Wray plants. Values are means ± SE of N = 6, and an asterisk indicates significantly different means between the two lines at *p* ≤ 0.05. Macia = black bars, and Wray = white bars

As expected for a grain cultivar, a higher grain yield was observed for Macia as compared to Wray (Fig. 3). Although Wray produced higher numbers of panicles per main stem (Fig. 3c), Macia produced larger and heavier panicles (Fig. 3a, d), larger seeds (Fig. 3b), higher total seed weight (Fig. 3e), and greater total seed number (Fig. 3f) on the main panicle. Thus, compared to Wray, Macia deposited greater amounts of fixed carbon in the panicle, which were ultimately stored in the seeds.

### Wray accumulates greater amounts of sugar in the stem than Macia

**Fig. 4:**
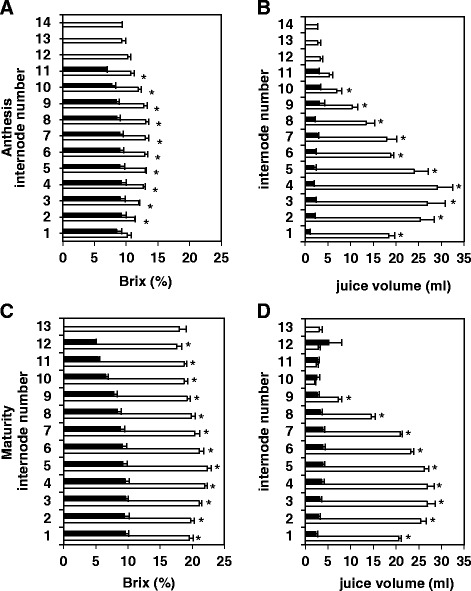
Percent Brix of individual internodes of Macia and Wray plants at different developmental stages. Individual internode number and percent Brix (**a**, **c**), and individual internode number and juice volume in ml (**b**, **d**) are shown at anthesis (**a-b**), and at physiological maturity (**c-d**). Values are means ± SE of N = 5, and an asterisk indicates significantly different means between the two lines at *p* ≤ 0.05. Macia = black bars, and Wray = white bars

Because the stem represents a strong sink for non-structural carbohydrates, we analyzed the total solute accumulation in Macia and Wray internodes, as measured in percent Brix. As shown in Fig. 4 and Additional file 2: Figures S1 and Additional file 3: Figure S2, Wray accumulated substantially higher Brix content compared to Macia at the whole-plant level, and also per internode for the great majority of internodes at most time points. At anthesis, the Brix content of the lowest internode (IN1) was not significantly different between the two cultivars. However, Wray displayed significantly higher Brix percentages in all of the other internodes (Fig. 4a). At maturity, Wray had accumulated approximately double to triple the Brix percentages in all internodes compared to Macia, with IN three to six exhibiting the highest amounts (Fig. 4c). Measurements of stem juice volume revealed that in Wray, the total volume did not change from anthesis to maturity, while in Macia, there was an increase of 37 % in the stem juice volume (Fig. 4b, d). However, despite the increase in stem juice volume for Macia, Wray stems exhibited approximately six to nine-fold higher juice volume than Macia. Hence, overall, the total solute accumulation was significantly higher in Wray stems compared with Macia.

### Sorghum stem phloem tissues are symplasmically isolated from surrounding storage parenchyma cells

**Fig. 5:**
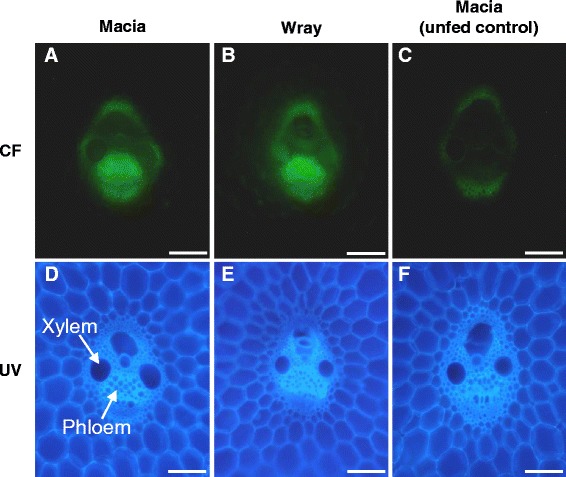
CF localization in stems of Macia and Wray plants fed CFDA from a source leaf. CF localized to the phloem tissue in the stem vasculature of Macia (**a**) and Wray (**b**). Control section from a Macia plant not fed CFDA (**c**). Only weak autofluorescence was detected. UV images of same sections to show cellular anatomy (**d**-**f**). Scale bar = 100 μm

The path that sucrose follows from the source leaves to storage within the stem sink tissues has not been conclusively determined for sorghum. To discern the path by which sucrose moves from the stem phloem to the storage parenchyma cells, we performed dye-loading studies using the phloem mobile dye carboxyfluorescein (CF). The membrane permeable, non-fluorescent diacetate form, CFDA, was applied to source leaves. Upon entering a cell, CFDA is converted into the fluorescent, membrane impermeable CF tracer, which is confined to the symplasm. The tip of leaf three or four, counting down from the panicle, was fed the CFDA solution for one hour. Plants were harvested after an additional five hours to allow the translocation of CF through the phloem into the stem tissues. If sucrose could follow a symplasmic path through plasmodesmata out of the phloem sieve tube into the stem parenchyma cells, we anticipated CF would likewise move along this route, and therefore be detected in the cytoplasm of the parenchyma cells. On the other hand, if the sucrose must be exported into the apoplasm across the sieve tube plasma membrane prior to entering into the parenchyma cells, we would anticipate that CF, a xenobiotic compound that presumably lacks endogenous transporters, would remain confined to the sieve tube. Examination of free-hand cross-sections of stem veins from both Wray and Macia showed that CF was detected strongly within the phloem cells (Fig. 5a, b). As shown by UV illumination, which outlines cellular anatomy through cell wall autofluorescence (Fig. 5d, e), the CF-containing cells were clearly identifiable as phloem cells. No CF signal was detected within the symplasm of the stem parenchyma cells, and only very slight CF signal was observed in their cell walls (Fig. 5a, b). This slight signal did not appear to result from CF localization within the cells, but more likely, the CF signal arose from within the phloem tissue, refracting through the cell walls of the stem, as well as potentially from CF being released from the phloem and contaminating the adjacent tissues during sample preparation. Hence, the signal appeared to be phloem specific. Control sections from plants not fed CFDA showed only weak autofluorescence from the cell walls of the xylem and the mestome sheath cells surrounding the vein (Fig. 5c, f). These data indicate that the sorghum stem phloem tissue is symplasmically isolated from the surrounding cells for both Wray and Macia, and hence, that the path of sucrose movement from the stem phloem to the storage parenchyma cells requires an apoplasmic transport step.

**Fig. 6:**
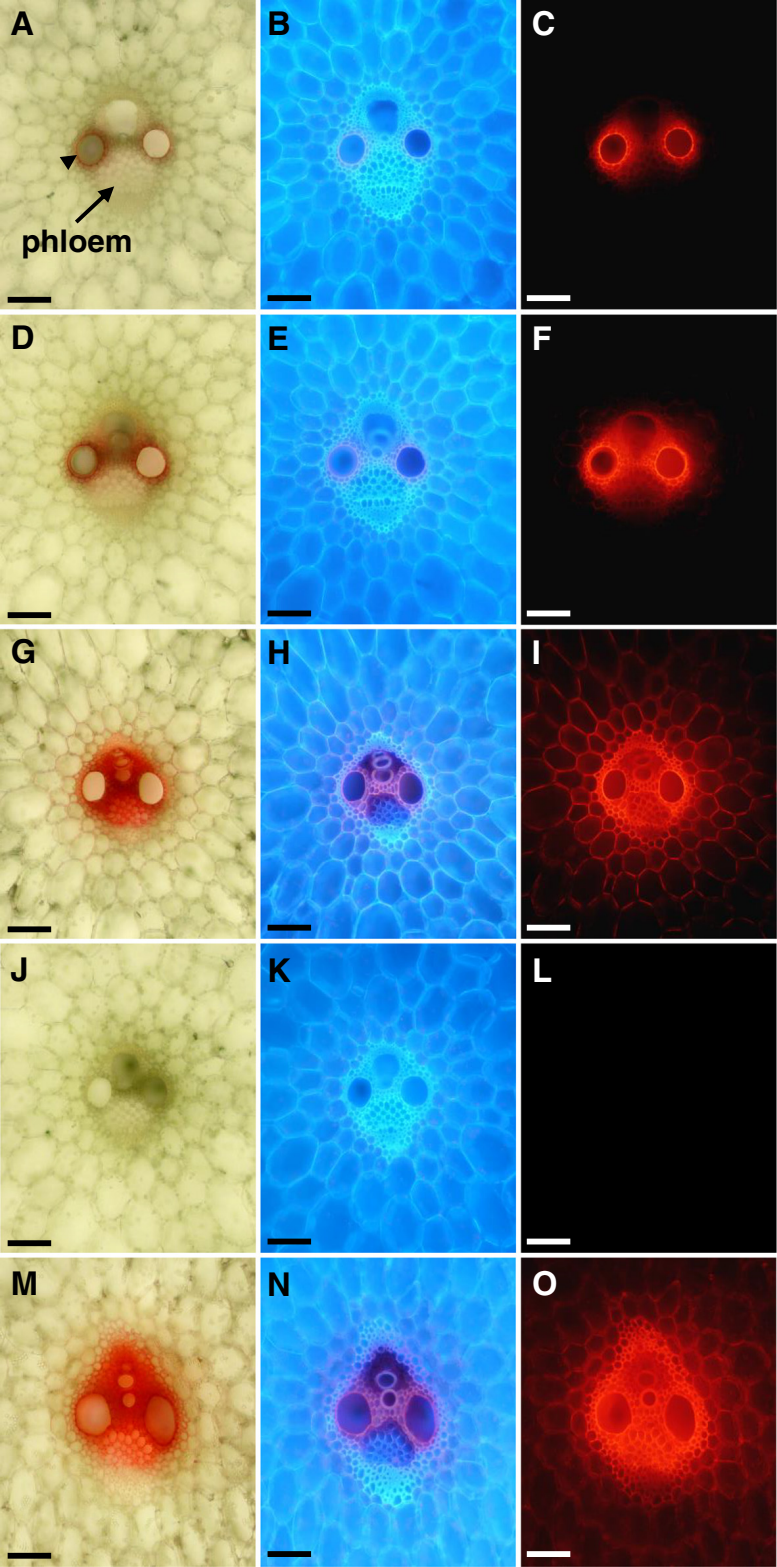
Safranin localization in a stem vein of Wray and Macia at post-anthesis. **a-l** correspond to Wray tissues, and **m-o** correspond to Macia tissues. Left, middle, and right columns represent transverse cross-sections of veins shown under bright-field, UV, and green light, respectively. Each row represents a single vein under the different types of illumination. Safranin was first detected in the walls of the xylem elements and adjacent xylem parenchyma cells, as indicated by the red coloration under bright-field (**a**; arrowhead) and by red fluorescence under green light (**c**). The safranin also showed some degree of fluorescence under UV illumination (**b**). Safranin was subsequently present throughout the cell walls of the xylem and was also detectable in the cell walls of the phloem adjacent to the xylem (**d-f**). Safranin eventually was observed in the cell walls throughout the vein and in the cell walls of the surrounding parenchyma cells (**g-i**). Control vein not fed safranin (**j-l)**. The same distribution of safranin was observed for a Macia stem vein (**m-o)**. Scale bar = 100 μm

To assess the ability of solute to move from the vein apoplasmic space to the storage parenchyma, Wray and Macia plants were fed from the base with safranin, a water soluble dye that stains lignin (Fig. 6). Safranin was initially detected in the cell walls of the xylem elements and those of the directly adjacent xylem parenchyma cells, as indicated by the red coloration under bright-field (Fig. 6a; arrowhead) and by red fluorescence under green light (Fig. 6c). Cellular anatomy within the region can be seen by the cell wall autofluorescence under UV illumination (Fig. 6b). Safranin also showed some fluorescence under UV light. With increased diffusion through the apoplasm, safranin was subsequently present throughout the cell walls of the xylem and was also detectable in the cell walls of the phloem cells adjacent to the xylem (Fig. 6d-f). Safranin eventually was present in all cell walls throughout the vein, and importantly, could be detected throughout the cell walls of the stem storage parenchyma cells outside of the vein (Fig. 6g-i). The red coloration and fluorescence were not observed in the control vein not fed safranin (Fig. 6j-l). The same apoplasmic distribution of safranin from the vein to the storage parenchyma cells was seen in Macia internodes (Fig. 6m-o). We observed no differences in dye transport studies comparing Wray and Macia, suggesting that any differences in sucrose accumulation between these cultivars is not likely due to differences in the unloading pathway used. Collectively, the CF and safranin dye transport studies suggest that for sucrose to move from the phloem to the stem storage parenchyma cells, sucrose must be effluxed across the sieve element plasma membrane, after which sucrose is able to diffuse within the apoplasm to the stem storage parenchyma cells, where it could be imported into the symplasm by a sugar transporter.

### Expression patterns of *SbSUTs* in mature leaf and stem tissues were generally similar between Macia and Wray

**Fig. 7:**
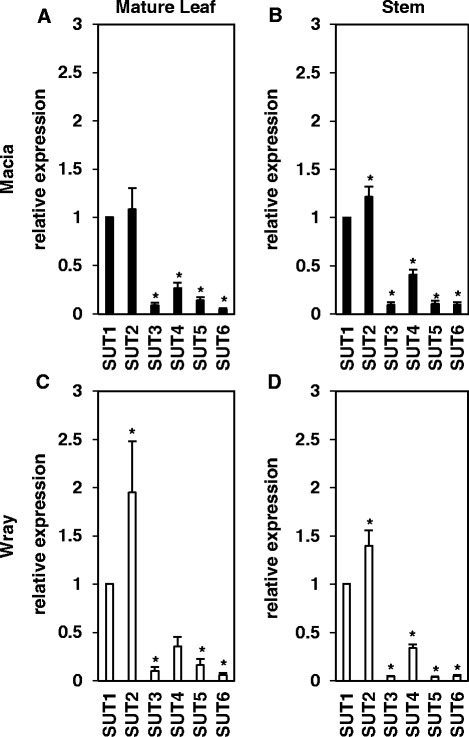
Expression levels of *SbSUT2-SbSUT6* relative to *SbSUT1* in Macia and Wray mature leaves and stems. **a**, **b** show Macia, and **c**, **d** show Wray; **a**, **c** are mature leaf tissues, and **b**, **d** are stems. Values are means ± SE of N = 5, and an asterisk indicates significantly different means between the two genes at *p* ≤ 0.05

Based on the results from the dye transport studies, we set out to investigate whether the *SbSUT* genes showed expression differences in the leaves and stems between Macia and Wray by quantitative RT-PCR (Fig. 7). Interestingly, we found that the overall expression pattern was similar in both cultivars and tissues, with *SbSUT3, SbSUT5,* and *SbSUT6* showing the lowest expression of the six genes. The Cq values calculated for these three genes were all above 31 (Additional file 4: Table S2). High Cq values indicate that gene expression levels are quite low, and this in turn causes high variation in accurately measuring expression and reduces the reproducibility of results [90–92]. Because the expression levels of *SbSUT3*, *SbSUT5*, and *SbSUT6* were exceedingly low in both cultivars and tissues, the subsequent analyses focused only on the relative expression levels of *SbSUT1, SbSUT2*, and *SbSUT4,* which were expressed in these tissues at appreciable and readily quantifiable levels (Additional file 4: Table S2).

**Fig. 8:**
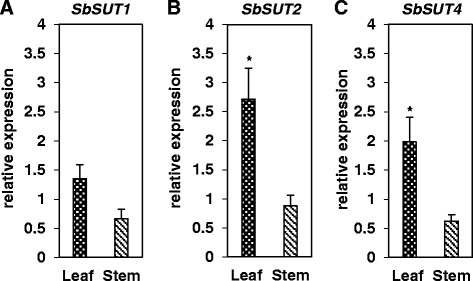
Expression levels *SbSUT1, SbSUT2* and *SbSUT4* in leaves and stems of Wray relative to Macia. Expression levels are shown for *SbSUT1* (**a**)*, SbSUT2* (**b**) and *SbSUT4* (**c**) An asterisk indicates significantly different means between the two lines at *p* ≤ 0.05 of N = 5. Leaf = black bars with white dots, and stem = hatched bars

In Macia, the expression of *SbSUT2* was similar to *SbSUT1* in mature leaves, but was significantly increased relative to *SbSUT1* in the stem (Fig. 7a, b). Meanwhile, the expression of *SbSUT4* was significantly lower than that of *SbSUT1* in both tissues. Similarly, in Wray, the expression of *SbSUT2* was significantly higher than that of *SbSUT1* and *SbSUT4* in both tissues, and that of *SbSUT4* was significantly lower than *SbSUT1* in stem but not leaf tissues. These data suggest that *SbSUT1*, *SbSUT2*, and *SbSUT4* may play roles in sucrose phloem loading in leaves and retrieval in stems (Fig. 7c, d). On the other hand, the relative expression of *SbSUT1* in both mature leaf and stem tissues was not significantly different between the two cultivars (Fig. 8a). Further, while the relative expression in the stem tissues of *SbSUT2* and *SbSUT4* was not different between the two cultivars, their expression was significantly higher in leaf tissue in Wray compared to Macia (Fig. 8b, c). In summary, no changes in expression levels in *SbSUT1, SbSUT2*, or *SbSUT4* were detected between Wray and Macia stem tissues, or for *SbSUT1* in mature leaf tissues, suggesting that differences in their expression at the RNA level do not contribute to differences in sucrose allocation. However, *SbSUT2* and *SbSUT4* displayed 2 to 2.5-fold higher expression in Wray mature leaf tissues relative to Macia, consistent with the hypothesis that these genes may play a role in sucrose partitioning and export differences between sweet and grain sorghum source leaves.

### Wray displays higher photosynthetic performance than Macia

**Fig. 9:**
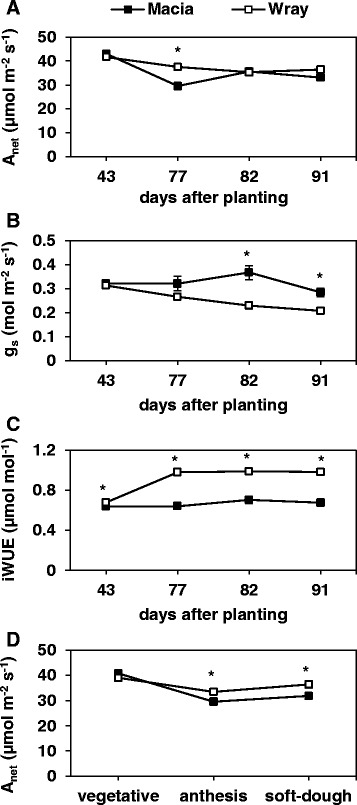
Differences in gas exchange parameters between Macia and Wray plants. Leaf net photosynthesis (A_net_) (**a**), Stomatal conductance (g_s_) (**b**), and instantaneous water-use-efficiency (iWUE) (**c**) at different days after planting. Leaf net photosynthesis at different developmental stages: vegetative, anthesis, and soft-dough (about 50 % of the grain weight has accumulated) (**d**). Values are means ± SE of N = 15, and an asterisk indicates significantly different means between the two lines at *p* ≤ 0.05. Macia = black squares, and Wray = white squares

The large differences in plant biomass, stem juice volume and Brix percentage, seed production, and time to anthesis indicate that the two sorghum cultivars employ different strategies for whole-plant carbohydrate partitioning. One possible contributor to this difference could be an increase in source strength of Wray compared to that of Macia. To investigate this hypothesis, we measured a number of photosynthesis-related parameters and compared them between the two cultivars (Fig. 9, Additional files 5: Figure S3, Additional file 6: Figure S4 and Additional file 7: Table S3). Wray and Macia leaf net photosynthesis (A_net_), stomatal conductance (g_s_), and instantaneous water use efficiency (iWUE) were assayed (Fig. 9). The only significant difference between Wray and Macia in A_net_, was seen at 77 DAP, with Wray having a higher rate than Macia (Fig. 9a). Similarly, stomatal conductance was not significantly different between the varieties at 43 and 77 DAP, but Macia showed higher values at 82 and 91 DAP compared to Wray (Fig. 9b). The significant increase in stomatal conductance in Macia was probably due to the significant increase in the number of stomata compared to Wray (Additional file 5: Figure S3). The increase in g_s_ was reflected in the decrease in iWUE of Macia (Fig. 9c). It is worth noting that because anthesis occurs at different times between the two cultivars (Fig. 1c), at 77 DAP, Wray was still at the vegetative stage while Macia was at anthesis. However, by analyzing the data at the comparable developmental stage, there were no differences between the two lines at the vegetative stage, but Wray displayed higher A_net_ rates at both anthesis and at soft-dough stages (Fig. 9d). There were no differences between the cultivars in photosynthetic rate in response to different light levels (Additional file 6: Figure S4) or in the maximum photochemical efficiency of photosystem II (Additional file 7: Table S3). Overall, our results indicate that Wray exhibited higher leaf photosynthetic performance after anthesis compared to Macia, and this difference in leaf source strength could play a role in the observed increase in biomass accumulation, stem juice volume, and Brix percentage exhibited by Wray.

## Discussion

Macia and Wray exhibited pronounced differences in their growth habits and carbohydrate partitioning patterns. We examined plant height, time to flowering, stem juice volume, Brix percentage, solute transport paths within mature stem tissues, *SbSUT* expression, and other attributes to understand the physiological and molecular differences in biomass and solute accumulation between Wray and Macia. Principally, we wanted to define the key parameters underlying how sweet sorghum accumulates high amounts of sugar in the stem, and to test the hypothesis that differences in *SbSUT* gene expression levels are responsible for the differential sugar accumulation in sweet vs. grain sorghum.

Wray and Macia differed for several traits related to stem biomass, juice volume, and solute content. Wray had a longer vegetative growth phase, with a concomitant expanded source strength, and showed an increased number of stem internodes. Internodes in Wray were significantly longer than those in Macia, and accounted for the differences in plant height between the cultivars. Although Macia had a shorter, more compact stature, it possessed thicker internodes, likely to support the greater weight of the panicle. The internodes in Wray were also significantly heavier than those in Macia in regards to both fresh and dry weights. The longer and heavier internodes of Wray contributed more to a higher juice volume of the stem compared to the shorter and wider internodes of Macia. Interestingly, in the comparisons of the Brix percentage and juice volume over developmental time, Macia showed only a small increase in juice volume and no changes in Brix values between anthesis and physiological maturity, reflecting the partitioning of carbohydrates to the panicle. Wray also showed no change in juice volume during this time period. However, Wray showed a highly significant increase in Brix percentages between anthesis and physiological maturity, indicating increased carbohydrate partitioning to the stem. Overall, on a per plant basis, Wray exhibited an approximately 24-fold greater abundance of stem solutes than Macia (~six-fold greater Brix levels multiplied by ~ four-fold more juice per stem). Hence, these data suggest that Wray accumulates higher stem solutes than Macia through a combination of increased internode number and length, higher juice volume per internode, and a greater sugar content per internode that occurs because of significant increases in sugar content during maturity, with the final component contributing the greatest effect.

### Superior photosynthesis-related parameters may account for greater solute accumulation in Wray’s stems

Photosynthesis, the biochemical process that drives carbon fixation in source leaves, could be one of the mechanisms underlying the striking differences in the abundance and partitioning of carbohydrates between Wray and Macia. Under optimal growth conditions, as shown by elevated CO_2_ concentration experiments, enhancing photosynthesis results in increased carbon supply and subsequent enhanced growth [27, 93–96]. Thus, the increased concentration of sugars in the Wray stem could be due, at least in part, to increased source strength compared to Macia. To test this hypothesis, we evaluated a number of photosynthesis-related parameters at different DAP and at different developmental stages. At the vegetative stage, there was no difference in the photosynthetic performance between Wray and Macia. The maximum photochemical efficiency of Photosystem II, net photosynthesis, and response of photosynthesis to increasing light intensity were also similar between the two cultivars. In contrast, with the start of the reproductive stage, Wray displayed higher rates of photosynthesis, reflecting a stronger source capacity that probably resulted in increased carbon availability and export to sink (stem) tissues. At 77 DAP, Wray stems started to accumulate higher concentration of solutes compared to Macia, with the exception of the top internode (Additional file 3: Figure S2). This increase could be attributable to the 21 % higher rates of photosynthesis in Wray compared to Macia. Measurements of photosynthesis at later dates did not show any differences between the two cultivars; however, a comparison of the plants at the same developmental stages revealed that Wray had an 11.7 % and 12.6 % higher rate of photosynthesis than Macia at the anthesis and soft-dough stages, respectively. Because of the high concentration of solutes in Wray stems, the increased photosynthesis in this cultivar might indicate a reduced sensitivity to feedback inhibition of photosynthesis by sugars, and/or an increased rate of sucrose export from the leaves [26, 97, 98]. The greater expression of *SbSUT2* and *SbSUT4* in Wray leaves could contribute to an increased rate of sucrose export (see below).

When Wray and Macia were measured at different DAP, the highest differences in solute concentration between the two cultivars occurred at 82 and 91 DAP. However, no differences in leaf photosynthesis were detected between the two cultivars at these time points. Interestingly, Wray displayed a significantly lower number of stomata on both leaf epidermal surfaces, causing decreased rates of stomatal conductance and thereby resulting in an increased iWUE. Hence, the differences in solute concentration in Wray stems could be attributable to the increased iWUE resulting in higher water availability for transport and storage of solutes. This increase is probably the largest contributor to the approximately six to ten-fold increased stem juice volume in Wray compared to Macia.

At all time points analyzed, Wray showed a statistically increased iWUE compared with Macia. From 43 DAP onwards, Wray also began to exhibit increased plant height and biomass. It would be interesting to monitor the growth of the root system in Wray and Macia over the growing season to determine if the differences in shoot biomass are mirrored by increased root biomass in Wray, which would be consistent with the hypothesis of greater source strength and increased carbon allocation to the roots. If so, the greater stem juice volumes measured in Wray may be explained at least partly by greater water uptake from the soil by a larger root system and a higher iWUE. A corollary of this idea is that a greater root mass in Wray necessitates greater sucrose export from the leaves to deliver sufficient fixed carbon to the roots to elaborate the larger root system. Coupled with the 24-fold higher sugar content in the stem, these data would suggest that Wray has much higher source strength than Macia. On the other hand, if Macia had the larger root system, it would reveal that it allocates a greater amount of carbon belowground, and that changes in root:shoot partitioning may help explain the differences in carbon allocation between the cultivars.

### Dye transport studies and the path of sucrose movement in sorghum stems

Sweet sorghum stems store vast amounts of soluble sugars within the storage parenchyma cells; however, the path that sucrose follows from the phloem to the terminal sink tissues is not known. Previous research examining sucrose catabolizing enzyme activities and stem-infused radioactive sucrose accumulation patterns found evidence that sucrose may initially follow a symplasmic path into a growing internode, but later at maturity switches to include an apoplasmic transport step, although its location along the transport path was not defined [76, 77, 79]. To characterize the route of sucrose movement, we performed dye transport studies, examining elongated internodes at the stage when sucrose begins to significantly accumulate within stems. Loading CF into the phloem of source leaves, and monitoring its location after transport into stem tissues, we determined that CF is confined to the phloem symplasm. This result suggests that the phloem sieve tubes in sorghum stems are symplasmically isolated from the surrounding cells, and that sucrose does not diffuse out of the phloem via plasmodesmata into adjacent cells. Hence, this implies that sucrose must be exported across the sieve element plasma membrane, and therefore, that phloem unloading in mature sorghum stem tissues occurs apoplasmically.

The hypothesized path for sucrose movement in sorghum stems contrasts with the proposed route for sucrose transport in sugarcane internodes, whereby sucrose unloads from the phloem symplasmically and moves entirely through the symplasm via plasmodesmata to the storage parenchyma cells [26, 58–60]. Sugarcane and sorghum are closely related grasses within the Andropogoneae tribe, and are thought to have diverged from a common ancestor approximately 10 million years ago [99]. Hence, it is somewhat surprising that the path for sucrose phloem unloading would be different between them, although it must be kept in mind that both are domesticated crops that have undergone strong selection for sugar accumulation; therefore, it is possible that strong selection pressures could have resulted in sugarcane using symplasmic phloem unloading to accumulate sugars to high levels within the stem, whereas sweet sorghum may have been selected to use apoplasmic phloem unloading to facilitate sugar accumulation in stems. It would be interesting to investigate the pathway of sucrose phloem unloading in grasses related to the common ancestor of sugarcane and sorghum that do not hyperaccumulate sucrose in their stems to understand the evolution of this trait.

To determine the path that sucrose could take once released into the phloem apoplasm in sorghum stems, we monitored the movement of safranin transport through the xylem. The xylem transpiration stream and the phloem apoplasm are continuous [100–102]; hence, safranin movement from the xylem shows where solutes in the phloem apoplasm are able to diffuse. We observed that safranin was able to diffuse through the cell walls of the stem vein to the apoplasm of the storage parenchyma cells. This indicates that sucrose could diffuse from the phloem apoplasm directly to the storage parenchyma cells, and that sucrose uptake and symplasmic transport are not required for sucrose to reach the storage parenchyma. Upon reaching the parenchyma cells, sucrose may be imported into the cells and stored within the vacuole [78]. The safranin movement data suggest that the lignified/suberized cell walls of the mestome sheath surrounding the vein are not an absolute barrier that prevents solute diffusion in sorghum. Similarly, it was previously reported that water, lanthanum, and ferrous ions were able to diffuse along the compound middle lamella of the suberized/lignified radial cell walls of the bundle sheath cells within a maize leaf, demonstrating a path for transpirational water movement from the xylem out to the mesophyll and epidermal cells [100]. This suggests that solutes such as sucrose within the apoplasmic fluid of sorghum veins could potentially move via the middle lamella of mestome sheath cell walls from the phloem apoplasm to the apoplasm of the stem parenchyma cells without entering the symplasm, thereby alleviating the need for SUT function.

### Proposed functions of *SbSUTs* in leaves and stems

From qRT-PCR expression experiments, we determined that *SbSUT1*, *SbSUT2*, and *SbSUT4* are expressed in mature leaves and internodes, whereas *SbSUT3*, *SbSUT5*, and *SbSUT6* are expressed at very low levels in these tissues. These data suggest that *SbSUT3*, *SbSUT5*, and *SbSUT6* unlikely have a major function in sucrose phloem loading in source leaves or sucrose partitioning in stems. Our findings on *SbSUT3* are similar to the undetectable expression observed for this gene in various tissues, as previously reported [75, 85, 86]. Likewise, Qazi et al. and Milne et al. did not detect the expression of *SbSUT5* in internodes and source leaves, respectively, while Shakoor et al. observed only very low *SbSUT5* expression across multiple sweet and grain sorghum tissues throughout development. Of interest, the present results for *SbSUT5* and *SbSUT6* differ in several respects from a previous report by Milne et al. [85]. These authors reported that *SbSUT5* was expressed in elongated stems at anthesis and suggested it might function in sucrose phloem unloading or retrieval within this organ. They also reported that *SbSUT6* was expressed in source leaves and only at low levels in elongated internodes, and suggested it might perform sucrose phloem loading in leaves. Several possibilities might explain the minor differences in gene expression (low vs. undetectable) and the few discrepancies between the present findings and those of previous studies. First, the sweet and grain sorghum cultivars accessed by Qazi et al., Milne et al., and Shakoor et al. are different from those analyzed in the present report. Hence, genotypic differences between our lines and their lines could account for the *SbSUT* expression differences [75, 85, 86]. Second, we collected mature leaf and stem tissues from field-grown plants, while other studies used glasshouse-grown plants. Thus, environmental differences might also partly explain the differing results [85, 86]. Milne et al. was the only other study that used qRT-PCR to measure *SbSUTs* gene expression. Unfortunately, these authors did not report the Cq values, and therefore it is possible that the raw expression levels were similar between the two studies, but that the values in the present study were considered to be below the threshold for biological significance. We recognize that from whole-tissue measurements, it is impossible to determine whether a gene may be highly expressed and function in a limited number of cells within a tissue, which could result in a high Cq value. Future genetic experiments to determine the biological functions of these *SbSUTs* through characterizing loss-of-function mutant plants are required to ascertain if these genes play a significant role in sucrose partitioning in sorghum leaves and stem tissues.

Based on their expression levels, *SbSUT1*, *SbSUT2*, and *SbSUT4* are thought likely to have biological roles in mature leaves and stem tissues. All three of these genes were more highly expressed in Wray than in Macia leaves, with statistically significant differences observed for both *SbSUT2* and *SbSUT4*. Although all three genes were also more highly expressed in Macia than in Wray stems, the differences were not statistically significant. These results suggest that differences in the expression of *SbSUT2* and *SbSUT4*, but not *SbSUT1*, could contribute to differences in sucrose phloem loading in mature leaf tissues between Wray and Macia, whereas these three genes probably do not contribute to differences in stem sucrose partitioning, at least based on RNA expression levels.

Our expression data for these three genes show some differences with previous findings. Unfortunately, because of confusing nomenclature, the genes we refer to as *SbSUT2* and *SbSUT4,* based on homology with the rice sequences [39], are inversely named in the Qazi et al. and Milne et al. references. For clarity in making comparisons between the data, we refer to these genes using our naming scheme (see Additional file 8: Table S4 for gene accession numbers). Because Qazi et al. did not perform qRT-PCR, we cannot directly compare their expression data with the present results. However, based on 35 cycles of RT-PCR, these authors reported that *SbSUT1* and *SbSUT2* had modestly lower expression in the internodes of their sweet sorghum variety compared with the grain sorghum during the early grain filling stage. No differences were observed in *SbSUT4* expression. Meanwhile, Milne et al. found that *SbSUT1* was substantially more highly expressed in source leaves and modestly higher in mature internodes of grain vs. sweet sorghum, whereas *SbSUT2* was not differentially expressed in source leaves and was hardly expressed in mature internodes. *SbSUT4* RNA was only approximately two-fold increased in grain sorghum leaves and similarly elevated in sweet sorghum internodes. Possible reasons for the differences between their findings and ours are noted above.

What might be the potential functions of *SbSUT1*, *SbSUT2*, and *SbSUT4*? In maize, *ZmSUT1* has been shown to function in sucrose phloem loading in leaves [82–84]. The lack of expression differences in the sorghum ortholog, *SbSUT1*, between sweet and grain sorghum suggests that its function in sucrose phloem loading is conserved, and that it does not likely underlie differences in carbohydrate partitioning between the cultivars. The biological functions of *SbSUT2* and *SbSUT4* remain to be determined. Based on homology with the related rice OsSUT2 and barley HvSUT2 proteins [43, 44], and the presence of a conserved tonoplast targeting motif in the encoded protein [42], we hypothesize that SbSUT2 localizes to the vacuole membrane and functions to export sucrose from the vacuole to the cytoplasm. The statistically significant higher expression of *SbSUT2* in the mature leaves of Wray compared with those of Macia may suggest that Wray is better able to efflux transitory sucrose stored in the vacuole of leaf cells. However, this hypothesis will need to be assessed experimentally. *SbSUT2* expression was not different in Wray and Macia stem tissues, suggesting that expression differences in this gene do not account for differences in stem sugar storage between the two cultivars.

*SbSUT4* has been shown to have sucrose transport activity by heterologous expression in yeast [85] and to be constitutively expressed [75]. The protein is hypothesized to be localized to the plasma membrane, and related group 3/type IIa SUTs have been proposed to function as sugar sensors or low affinity/high capacity sucrose transporters [15, 17, 39, 42, 103]. Loss-of-function mutations in the orthologous gene in *Arabidopsis thaliana* did not condition any apparent morphological or carbohydrate partitioning-related phenotypes [104]. Based on RNA expression, *SbSUT4* is more highly expressed in Wray leaves than in Macia, and may contribute to greater sucrose phloem loading capacity. Hence, in summary, *SbSUT2* and *SbSUT4* are the only abundantly expressed sorghum *SUT* genes that exhibited differential RNA expression in mature leaves and may contribute to differences in source strength in Wray as compared with Macia. None of the sorghum *SUT* genes showed significant differential gene expression in stem tissues between Wray and Macia. These data suggest that, based on RNA expression, the function of *SbSUT* genes are unlikely to explain the differences in stem sugar accumulation between these cultivars.

An additional possibility to explain the differences in carbohydrate partitioning between Wray and Macia is differential sink control of phloem transport and unloading. At the flowering stage, the two major competing sinks in sweet and grain sorghum are the stem and developing panicle. We did not analyze *SbSUT* gene expression in developing panicles, and therefore our discussion is limited to maturing stem tissues. Here, we found significant differences in sugar accumulation in stem tissues between Wray and Macia. In Wray, stem solute content increased approximately 24-fold during the time from anthesis to physiological maturity, indicating that the stem sink tissues were actively accumulating sugars. Additionally, although we observed no differences in *SbSUT* expression in stem tissues, we found that *SbSUT2* and *SbSUT4* were upregulated in the source leaves of Wray as compared to Macia. Similarly, we observed increased photosynthesis in the source leaves at 77 DAP in the former relative to the latter. These data suggest that the strong stem sink in Wray might upregulate both carbon assimilation and the phloem loading of sucrose within the leaves to increase sucrose delivery to the stem internodes. Precedence for this hypothesis is provided by experiments in which the modification of sink strength led to altered *SUT* gene expression. For example, the feeding of sucrose to the leaf transpiration stream of sugar beet (*Beta vulgaris* L.) was used to mimic decreased sink demand, and resulted in decreased *BvSUT1* expression and activity*,* which would consequently result in diminished phloem sucrose loading in source leaves [105]. Similarly, increasing sink demand has been found to regulate *SUT* expression and thus to control sucrose transport. Zhou et al. tested the hypothesis that sink demand for carbon skeletons regulates the activities of sucrose transporters in developing pea (*Pisum sativum* L.) cotyledons [29]. The authors found that sucrose influx into developing pea cotyledons acted as a signal that upregulated *PsSUT1* expression, thereby linking sink demand for assimilates with sucrose import. Finally, in sugarcane, through shading all but one source leaf to increase relative sink strength, it was found that decreased stem sucrose concentration correlated with an increase in phloem transport from the leaf and an enhanced rate of photosynthesis [27]. These findings suggest that the strong stem sink tissues in Wray may use similar mechanisms to control phloem transport and carbohydrate partitioning to the stem.

### Model for sucrose movement from the phloem to storage parenchyma cells in sorghum stems

**Fig. 10:**
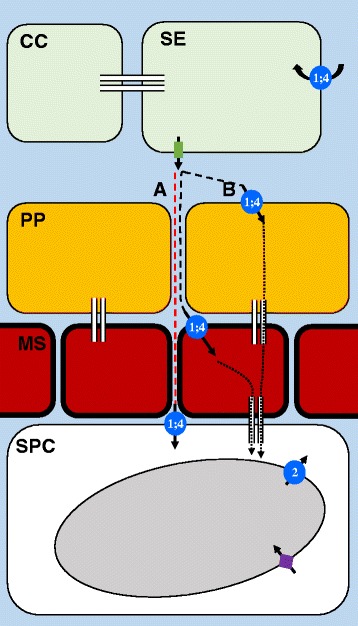
Model for sucrose movement from stem phloem to storage parenchyma cells in sorghum. The phloem companion cells (CC) and sieve elements (SE) are shown in green; the phloem parenchyma (PP), in orange; the mestome sheath (MS) cells, in red; and storage parenchyma cell (SPC), in white. The apoplasmic space is indicated by the light blue background. A vacuole in the SPC is shown in grey. Plasmodesmata are shown by thin, white rectangles. The red dashed line (**a**) indicates an entirely apoplasmic path for sucrose. The black dashed lines (**b**) indicate sucrose initially moving through the apoplasm, followed by import into either the PP or MS cells, and the black dotted lines represent sucrose symplasmic movement. SUT proteins are shown by a blue circle, with an arrow indicating the direction of sucrose uptake, and the numbers represent SbSUT1 and/ or SbSUT4 (1;4), or SbSUT2 (2). Green rectangle with an arrow indicates a SWEET sucrose effluxer, and purple diamond with arrow refers to TST protein located on the tonoplast

From the dye loading and *SbSUT* expression data, we propose the following model for sucrose unloading and storage in sorghum stem tissues (Fig. 10). Because the phloem sieve tubes are symplasmically isolated from surrounding cells based on the CF transport studies, it suggests that sucrose must be effluxed across the sieve element/companion cell plasma membrane. We hypothesize that this function is mediated by SWEET efflux proteins, since the pH gradient across the sieve element plasma membrane, and the protein topology and H^+^/sucrose co-transport activity of SUTs predict sucrose uptake from the apoplasm rather than release. Therefore, we conclude that *SbSUTs* do not likely function in sucrose phloem unloading in Wray or Macia stems, but instead function in sucrose retrieval into the phloem sieve tube to help maintain the osmotic gradient between distant source and sink tissues, and/or in sucrose uptake into surrounding cells. Upon efflux to the apoplasm, sucrose is able to migrate into the cell walls of the storage parenchyma cells, as indicated by the safranin diffusion (Fig. 10a). SWEETs, SbSUT1, and/or SbSUT4 may function to uptake sucrose into cells along the post-phloem unloading path (e.g., mestome sheath) or directly into the stem storage parenchyma. If sucrose is uptaken by the phloem parenchyma or mestome sheath cells, it presumably moves through plasmodesmata to the stem storage parenchyma cells (Fig. 10b). Importantly, the presence of an apoplasmic transport route for safranin, and potentially sucrose, from the phloem apoplasm to the stem storage parenchyma cells does not indicate that this pathway contributes a significant amount to the overall accumulation of sucrose within stem tissues. For example, immediate uptake by cells outside of the sieve element/companion cell complexes might account for the majority of uptake from the apoplasm. In addition to an assessment of the rate at which sucrose can move along the apoplasmic path to the stem parenchyma cells, future studies will also need to determine whether 1) heterogeneity is present in sucrose phloem unloading in sorghum stems from different cultivars [106], 2) a developmental shift occurs from one path to the other [12, 57], and 3) one pathway predominates over the other for sucrose accumulation in sweet sorghum stems.

For the storage parenchyma cells, we predict that sucrose is transported into the vacuoles by TSTs [52, 107, 108], since these transporters have been previously shown to be expressed in sorghum stem tissues [75, 109]. If sucrose is exported from the vacuoles, SbSUT2 may perform this role. Based on this model, a tantalizing possibility is that a key step in sucrose accumulation in the sweet sorghum stem is controlled by a TST, as has been recently reported for the control of sucrose storage in sugar beet [52]. While sugarcane and sweet sorghum may have been selected to utilize different pathways for sucrose phloem unloading from the stem, it will be of exceeding interest to examine the function of TSTs in sweet sorghum and sugarcane to determine if the same gene function has been convergently selected during domestication of the world’s major sugar crops. Future research will investigate this possibility.

## Conclusions

Sugar accumulation within the stem of sweet sorghum compared to grain sorghum is not due to differences in the phloem unloading pathway. Moreover, the stem phloem tissue is symplasmically isolated from surrounding cells, suggesting that sucrose is unloaded apoplasmically. The lignified mestome sheath does not prevent apoplasmic diffusion from the phloem apoplasm to the stem storage parenchyma cell walls, suggesting sucrose may follow an apoplasmic path. These results suggest that the path of sucrose movement in sorghum stems differs from sugar accumulation within the stems of the closely related grass, sugarcane. Additionally, differences in stomatal density, water use efficiency, and carbon assimilation may contribute to a greater mature leaf source strength in Wray compared with Macia. Finally, based on RNA expression levels, *SbSUTs* are unlikely to contribute to sugar accumulation differences between Wray and Macia stems, but *SbSUT2* and *SbSUT4* could play roles in Wray’s increased leaf source strength.

## Methods

### Plant materials and growth conditions

Seeds of the sweet sorghum (*Sorghum bicolor* L. Moench) cultivar Wray and grain sorghum cultivar Macia were kindly provided by Dr. Ismail Dweikat at the University of Nebraska-Lincoln. Plants were grown at the South Farm Research Center at the University of Missouri, Columbia, MO, USA (38°54’N, 92°16’W) during the summers of 2013 and 2014. Seeds were planted in three randomized experimental blocks (blocked by location and time), with each one containing a plot for each sorghum cultivar. Every plot was composed of four adjacent rows 6.6 m in length. The two outer rows served as buffers, while all measured parameters were taken from the plants in the two middle rows. A total of 100 seeds were planted in each row. At the three-leaf stage, the seedlings were hand-thinned to 20 individuals per row to provide a spacing of approximately 0.3 m between plants.

### Growth, biomass, and yield measurements

A number of biomass-related parameters were measured at different DAP, including the main stem height (measured from the soil surface to the base of the panicle) and leaf and tiller number per plant [110]. Additionally, the total leaf area (LA) and the length and width of the second leaf from the top of the plant (tLL and tLW), the leaf at mid-height (mLL, mLW), and the leaf at the plant base (bLL, bLW) were measured using a LI-3000 leaf area meter (LI-COR Inc., Lincoln, NE). The main stems were collected at physiological maturity (defined by the presence of the black layer over the hilium at the seed base), and the internodes (IN) were separated and weighed to determine the fresh weight (FW). The IN segments were oven-dried at 60 **°**C for five days to obtain the dry weight (DW). Main stem diameter was measured and used to calculate the stem circumference in the middle of three IN located at the base, middle, and top of the main stem.

Various grain yield components were measured after harvest. After the panicle number of the main stem was counted, the main panicles were harvested, dried, and weighed to determine the total weight. The main panicles were then carefully threshed to determine the total seed weight and number for each one.

### Brix content

Brix measurements, which indicate the amount of solutes in the juice, were determined directly after harvest. After removal of the panicles and leaves from the main stem, each internode was isolated and immediately processed. The juice from each segment was extracted by passing them through a bench top power mill Model SC-3 Sugarcane Juicer (Jucernet, Mulligan Associates Inc., Jupiter, FL, USA). When the stem sections were small, the juice was extracted by hand using a garlic press and filtered through a double layer of cheesecloth. Subsequently, the juice volume was measured using a graduated cylinder, and the Brix percentage was determined with 1 ml of juice using an Atago 3810 digital handheld pocket refractometer (PAL-1, Atago USA Inc., Bellevue, WA, USA). Brix measurements were recorded for plants at anthesis and physiological maturity. In addition, a separate set of plants was used to determine the total juice volume and Brix percentage at maturity using uncut main stems stripped of leaves and without panicles.

### Gas exchange and chlorophyll fluorescence measurements

Gas exchange measurements were taken on fully expanded source leaves using a portable infra-red gas exchange system (LI-6400XT, LI-COR Inc., Lincoln, NE, USA) at different DAP as described [111, 112]. Net photosynthesis (A_net_, μmol m^−2^ s^−1^) and stomatal conductance (g_s_, mol m^−2^ s^−1^) rates were measured at a photon flux density of 2000 μmol m^−2^ s^−1^ (determined from running an initial light response curve; Additional file 6: Figure S4) and ambient CO_2_ concentration of 400 μmol mol^−1^. Measurements were performed between 9:00 AM and 12:00 PM at 43, 77, 82, and 91 DAP on 15 plants per sorghum variety. Instantaneous water use efficiency (iWUE) was determined using the following equation:

1 $$ \mathrm{iWUE}\ \left(\upmu \mathrm{mol}\ {\mathrm{mol}}^{-1}\right)={\mathrm{A}}_{\mathrm{net}}\left(\upmu \mathrm{mol}\ {\mathrm{mol}}^{-1}{\mathrm{s}}^{-1}\right)/\mathrm{T}\ \left(\mathrm{mol}\ {\mathrm{mol}}^{-1}{\mathrm{s}}^{-1}\right) $$

where T is the transpiration rate.

The maximum photochemical efficiency of photosystem II (Fv/Fm) was determined on dark-adapted leaves using a leaf fluorometer attached to the LI-6400XT infrared gas analyzer. Chlorophyll fluorescence was measured on fully expanded source leaves, adapted to darkness for at least 30 min using dark-adapting clips at 56 DAP. A Minolta SPAD-502 meter (Spectrum Technologies, Plainfield, IL) was used to estimate chlorophyll content at 43, 77, 82, and 91 DAP [113].

### Stomatal counts

Epidermal leaf imprints were collected from the adaxial and abaxial leaf surfaces of plants at anthesis [114, 115]. After one drop of “Superglue” was applied onto the target leaf area, a clean glass slide was gently pressed against it and held firmly in place until the adhesive dried. The glass slide was lifted carefully from the leaf surface, which resulted in the direct placement of the epidermal impression onto the glass slide. Five plants were sampled per genotype, and three regions from the center of mature, fully expanded source leaves were collected on both epidermal surfaces. Images were taken under bright-field illumination using a Nikon Eclipse 80i compound fluorescent microscope at 20× magnification and analyzed using the open-source ImageJ software (http://rsb.info.nih.gov/ij/) to count the number of stomata per field of view.

### Dye transport assays

Feeding experiments using 5,6-carboxyfluorescein diacetate (CFDA, 50 μg/ml, Invitrogen, Carlsbad, CA, USA) were performed as previously described [36, 116]. Plants were grown in the fall and winter in a greenhouse supplemented with high-pressure sodium lighting (1600 μmols m^−2^ sec^−1^) to provide a 16-h photoperiod at a day/night temperature of 30/24 °C. Plants with fully elongated internodes just prior to or at anthesis were used for the experiments. The cut tip of the third or fourth mature source leaf below the panicle was submerged into a 30 ml CFDA solution for one hour. After five additional hours, free-hand transverse sections of the fully elongated stem tissue at multiple internodes below the fed leaf were mounted in water and examined with a Nikon Eclipse 80i epifluorescent microscope equipped with a 100-W mercury bulb using either UV (360- to 370-nm excitation filter and a 420-nm long-pass emission filter) or blue light (465- to 495-nm excitation filter and a 515- to 555-nm band-pass emission filter), respectively [117].

For the safranin O dye movement studies, post-anthesis sorghum plants were cut at the base of the stem, and the cut ends were immersed into a 0.07 % aqueous solution of safranin O for 2 h [118]. Transverse hand-cut stem sections were mounted in water and examined under the microscope using bright-field, UV, and green illumination (530- to 560-nm excitation filter and 590- to 650-nm band-pass emission filter), respectively.

All images were captured using a Nikon DXM1200F camera and Nikon NIS Elements F software (version 3.0). For each type of illumination, all photographs in a figure were taken using identical microscope and camera settings, including the same gain settings.

### Total RNA isolation and reverse transcription

For gene expression studies, mature source leaf and stem samples were collected from field-grown plants at anthesis, immediately frozen in liquid nitrogen, kept on dry-ice, and stored at −80 °C until processed. Leaf tissues were collected from the center of the leaf blade, omitting the mid-rib, and stem tissues were harvested from the middle part of the internode located at the mid-height of the main stem. Five biological replications per cultivar were used.

Leaf tissues were ground to a fine powder in liquid nitrogen using a pre-chilled mortar and pestle. To grind stem tissues, dry-ice was first ground in a cryogenic tissue grinder. The resultant dry-ice powder was discarded, and frozen stem fragments were immediately placed into the chilled grinder and processed. The powdered tissue was collected into frozen 15 ml tubes and placed on dry-ice until all samples were processed.

Total RNA was extracted from 100 mg of frozen ground tissue aliquoted into a frozen 1.5 ml Eppendorf tube using 1 ml of TRIzol® reagent (Invitrogen, Carlsbad, CA, USA) and 200 μl of chloroform. Afterwards, 175 μl of the supernatant was used for RNA isolation. The RNA was treated with RNase-free DNase (Qiagen, Valencia, CA, USA) and then isolated using the RNeasy MinElute Cleanup kit (Qiagen, Valencia, CA, USA). The RNA was dissolved in RNase-free water, and its quality was assessed by spectrophotometry and gel electrophoresis.

One μg of total RNA was used to synthesize the complementary DNA (cDNA) using iScript^TM^ Reverse Transcription Supermix (Bio-Rad, Hercules, CA, USA) according to the manufacturer’s protocol. To identify a suitable reference gene for normalization of *SbSUT* genes expression, the expression stability of several widely used endogenous reference genes, tubulin (Sb02g037260), ubiquitin (Sb02g021080), and actin (Sb01g010030), were tested, but their expression was unstable between leaf and stem tissues. Thus, an exogenous gene, luciferase, was used as the reference gene [119, 120]. Prior to cDNA synthesis, 50 pg of luciferase RNA (Promega, Madison, WI, USA) was spiked into the RNA to serve as the reference gene for the quantitative real-time reverse transcription-polymerase chain reaction (qRT-PCR) experiments.

### qRT-PCR amplification and data analysis

The primers used in this study are listed in Additional file 8: Table S4. The primers were designed to specifically amplify only the *SbSUT* gene of interest and to generate products between 100–150 bp in size for each *SbSUT* gene. The primer sequences were first subjected to Basic Local Alignment Search Tool (BLAST) against the sorghum genome (*Sorghum bicolor* v2.1 available at www.phytozome.net) to ensure a unique homology for the target sequences, and were ordered from Integrated DNA Technologies (Coralville, IA, USA). The PCR conditions were optimized across a temperature gradient to determine the best annealing temperature and to ensure a single PCR product was amplified. The PCR product was analyzed on a 2 % agarose gel to confirm it was a single product of the expected size. PCR products were purified using the RapidTip purification kit (Diffinity Genomics, West Henrietta, NY, USA) and sequenced to confirm the PCR primers were specific for the target gene. All *SbSUT* gene-specific primers were designed as described above, except for the *SbSUT5* primer set. This primer set was previously published by Milne et al. [85], and was also validated according to the above procedure.

A total of four technical replications for each of the five biological replicates were used in the qRT-PCR experiment, and nuclease-free water was used instead of cDNA in the non-template control. For each reaction, 10 ng of cDNA was mixed with 5 μl of ScoFast™ EvaGreen® Supermix with Low ROX (Bio-Rad, Bio-Rad, Hercules, CA, USA), and 0.4 μM of both the forward and reverse primers were added for a final volume of 10 μl. A CFX384 Connect^TM^ Real-Time PCR Detection System (Bio-Rad, Hercules, CA, USA) was used to run the analysis. The amplification program was as follow: 95 **°**C for 30 s followed by 40 cycles of 95 **°**C for 5 s, 57 **°**C for 30 s, and a final temperature increment of 0.5 **°**C for 5 s from 65 **°**C to 95 **°**C.

The qRT-PCR data was analyzed following the Pfaffl method [121]. First, the PCR efficiency for both the reference and target genes were determined using the following equation:

2 $$ \mathrm{E}=1{0}^{\left[-1/\mathrm{m}\right]} $$

where E is the PCR efficiency and m is the slope of the standard curve obtained by the qRT-PCR analysis of known serial dilutions of the cDNA. Afterwards, the relative expression ratio of the target gene was calculated based on E and the Cq deviation of the sample versus a control expressed in comparison to a reference gene as shown in the following equation:

3 $$ \mathrm{R}={\left({\mathrm{E}}_{\mathrm{target}}\right)}^{\Delta \mathrm{C}\mathrm{q}}{{}_{\mathrm{target}}}^{\left(\mathrm{control}-\mathrm{sample}\right)}/{\left({\mathrm{E}}_{\mathrm{ref}.}\right)}^{\Delta \mathrm{C}\mathrm{q}}{{}_{\mathrm{ref}.}}^{\left(\mathrm{control}-\mathrm{sample}\right)} $$

where R is the relative expression ratio, E_target_ is the PCR efficiency of the target gene (*SbSUTx*), E_ref._ is the PCR efficiency of the reference gene (luciferase), ΔCq_target_ is the Cq deviation of the control minus the sample of the target gene, and ΔCq_ref._ is the Cq deviation of the control minus the sample of the reference gene. A Cq value greater than 31 indicates that the gene expression level is below that which can be accurately measured [90–92]. The average of the fold change values from the five biological replicates was used to plot the expression level of the *SbSUT* genes in the two tissues for the two cultivars, and to plot the expression level of each *SbSUT* gene in Wray using the corresponding gene in Macia.

### Statistical analyses

Statistical analyses were performed using SAS (version 9.3, Cary, NC, USA; SAS Institute, Inc. 1998). The mixed model analysis of variance (PROC MIXED, SAS) was used to determine if significant differences were present between Wray and Macia, with the cultivar and plants used as the fixed and random effects, respectively. All results are reported as mean ± standard error (SE); significant differences between the cultivars were assessed at p ≤ 0.05.

## Additional files

Additional file 1: Table S1. Morphological parameters of Macia and Wray plants grown in the field and measured on different days after planting (DAP). Values are means ± SE of N = 5. Lowercase letters indicate significantly different means between the two lines at p ≤ 0.05^**a**^, p ≤ 0.01^**b**^, or p ≤ 0.001^**c**^. Abbreviations: MS LN, Main Stem Leaf Number; MS LA, Main Stem Leaf Area; tLL, top Leaf Length; mLL, middle Leaf Length; bLL, base Leaf Length; tLW, top Leaf Width; mLW, middle Leaf Width; bLW, base Leaf Width; TN, Tiller Number; SPAD is a relative measure of chlorophyll; ns, not significant. (PDF 70 kb) Additional file 2: Figure S1. Percent Brix (**A**) and total juice volume (**B**) of whole main stems harvested at physiological maturity. Values are means ± SE of N = 5, and an asterisk indicates significantly different means between the two lines at *p* ≤ 0.05. Macia = black boxes, and Wray = white boxes. (PDF 61 kb) Additional file 3: Figure S2. Percent Brix of top (**A**), mid-height (**B**), and basal (**C**) internodes sampled on different days after planting. Values are means ± SE of N = 15, and an asterisk indicates significantly different means between the two lines at *p* ≤ 0.05. Macia = black boxes, and Wray = white boxes. (PDF 55 kb) Additional file 4: Table S2. Average Cq values ± SE obtained for each *SbSUT* gene in both leaf and stem tissues of Wray and Macia. (PDF 87 kb) Additional file 5: Figure S3. Average number of stomata per field of view on the adaxial and abaxial leaf surfaces of field grown Macia and Wray plants. A total of N = 3 impressions were collected from each leaf surface of N = 5 plants per cultivar. Values are means ± SE, and an asterisk indicates significantly different means between the two lines for each surface or between opposite surfaces for each cultivar at *p* ≤ 0.05. Macia = black boxes, and Wray = white boxes. (PDF 51 kb) Additional file 6: Figure S4. Light response curves of Macia and Wray leaves from plants grown in the field, measured 64 days after planting. Net assimilation (A) is plotted on the y-axis, and photosynthetically active radiation (PAR) is plotted on the x-axis. Values are means ± SE of N = 5. Macia = black boxes, and Wray = white boxes. No statistical differences were detected between the two cultivars. (PDF 53 kb) Additional file 7: Table S3. Chlorophyll fluorescence (Fv/Fm) measured on dark adapted leaves of Macia and Wray plants grown in the field. Measurements were taken 56 days after planting on N = 5. No statistical differences were detected between the two cultivars. (PDF 88 kb) Additional file 8: Table S4. List of PCR primer sets used in the qRT-PCR analyses. (PDF 80 kb)
