# Supplementary material for: Sucrose accumulation in sweet sorghum stems occurs by apoplasmic phloem unloading and does not involve differential Sucrose transporter expression
Source: BMC Plant Biol. 2015 Jul 30;15:186. doi: 10.1186/s12870-015-0572-8 (PMC4518677; doi:10.1186/s12870-015-0572-8)
Supplement: Additional file 1: Table S1. — Morphological parameters of Macia and Wray plants grown in the field and measured on different days after planting (DAP). Values are means ± SE of N = 5. Lowercase letters indicate significantly different means between the two lines at p ≤ 0.05a, p ≤ 0.01b, or p ≤ 0.001c. Abbreviations: MS LN, Main Stem Leaf Number; MS LA, Main Stem Leaf Area; tLL, top Leaf Length; mLL, middle Leaf Length; bLL, base Leaf Length; tLW, top Leaf Width; mLW, middle Leaf Width; bLW, base Leaf Width; TN, Tiller Number; SPAD is a relative measure of chlorophyll; ns, not significant. (PDF 70 kb) [file 12870_2015_572_MOESM1_ESM.pdf]

Additional file 1: Table S1. Morphological parameters of field-grown Macia and Wray plants measured at different days after planting (DAP).

	43 DAP			77 DAP			82 DAP			91 DAP		
	**Macia**	**Wray**		**Macia**	**Wray**		**Macia**	**Wray**		**Macia**	**Wray**	
MS LN	10.2 ±0.2	10.6 ±0.2	*ns*	18.6 ±0.2	22.2 ±0.6	**c**	19 ±0.5	22.8 ±0.5	**c**	19.4 ±0.2	22.2 ±0.4	**c**
MS LA (cm^2)	872 ±83	664 ±47	*ns*	4442 ±79	4827 ±269	*ns*	4434 ±131	4758 ±253	*ns*	4578 ±156	4858 ±78	*ns*
tLL (cm)	37.7 ±1.1	43.1 ±1	**b**	48 ±2.1	20.8 ±1.1	**c**	48.1 ±2.7	22 ±1.9	**c**	43.3 ±2.7	23 ±2.1	**c**
mLL (cm)	33.9 ±1.7	33.3 ±1.5	*ns*	53.9 ±1.5	83.7 ±3.9	**c**	55.5 ±1.4	80.4 ±4	**c**	62.3 ±3	83 ±2.4	**c**
bLL (cm)	21.1 ±0.4	16.2 ±0.8	**c**	33.9 ±1.7	43.5 ±1.1	**b**	35.8 ±1.2	39.2 ±2	*ns*	34.6 ±2.1	44.1 ±2.6	***a***
tLW (cm)	6.5 ±0.3	5.5 ±0.1	**b**	8.9 ±0.3	2.1 ±0.2	**c**	8.6 ±0.5	2.9 ±0.2	**c**	7 ±0.2	2.6 ±0.2	**c**
mLW (cm)	4.9 ±0.2	3.6 ±0.3	**b**	9.2 ±0.2	6.4 ±0.2	**c**	8.9 ±0.3	6.7 ±0.5	**c**	8.4 ±0.2	6.6 ±0.2	**c**
bLW (cm)	1.7 ±0.1	1.9 ±0.3	*ns*	5.2 ±0.2	4.7 ±0.3	*ns*	5.3 ±0.1	4.6 ±0.3	***a***	5.4 ±0.3	5 ±0.4	*ns*
TN	1.2 ±0.2	3.8 ±0.4	**c**	1.2 ±0.2	4.8 ±0.6	**c**	1 ±0	4.3 ±0.3	**c**	1 ±0	3.4 ±0.2	**c**
SPAD	49 ±0.6	50 ±0.5	*ns*	64 ±0.5	56 ±0.4	**c**	62 ±1.1	57 ±0.5	**b**	65 ±0.9	57 ±0.6	**c**

Lowercase letters indicate significantly different means between the two lines at $p \leq 0.05^{a}$, $p \leq 0.01^{b}$, or $p \leq 0.001^{c}$. Abbreviations: MS LN, Main Stem Leaf Number; MS LA, Main Stem Leaf Area; tLL, top Leaf Length; mLL, middle Leaf Length; bLL, base Leaf Length; tLW, top Leaf Width; mLW, middle Leaf Width; bLW, base Leaf Width; TN, Tiller Number; SPAD is a relative measure of chlorophyll; ns, not significant
