# Supplementary figures and images for: Sucrose accumulation in sweet sorghum stems occurs by apoplasmic phloem unloading and does not involve differential Sucrose transporter expression
Source: BMC Plant Biol. 2015 Jul 30;15:186. doi: 10.1186/s12870-015-0572-8 (PMC4518677; doi:10.1186/s12870-015-0572-8)

**Additional file 2: Figure S1**

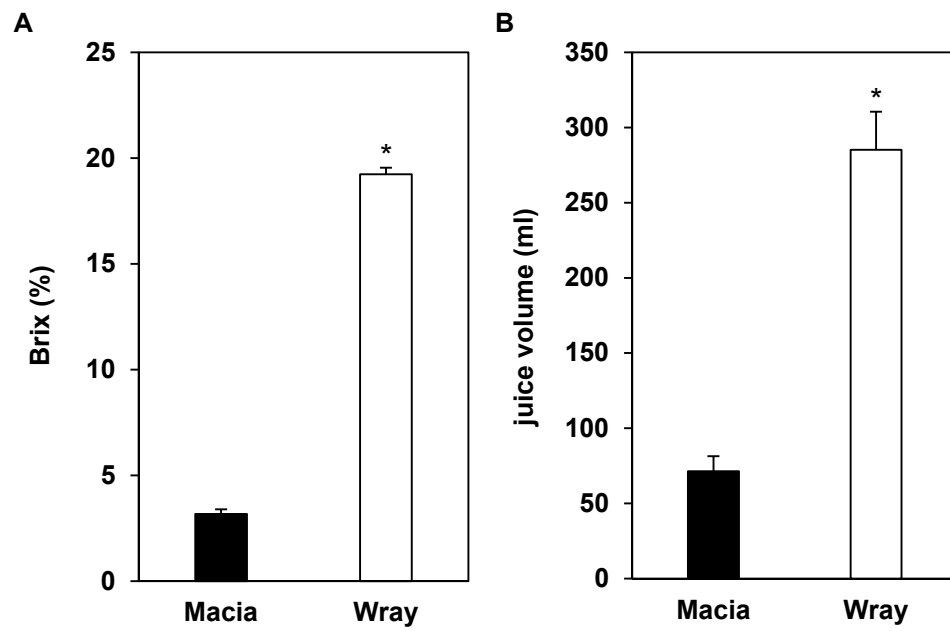

Supplement: Additional file 2: Figure S1. — Percent Brix (A) and total juice volume (B) of whole main stems harvested at physiological maturity. Values are means ± SE of N = 5, and an asterisk indicates significantly different means between the two lines at p ≤ 0.05. Macia = black boxes, and Wray = white boxes. (PDF 61 kb) [file 12870_2015_572_MOESM2_ESM.pdf]

Additional file 3: Figure S2

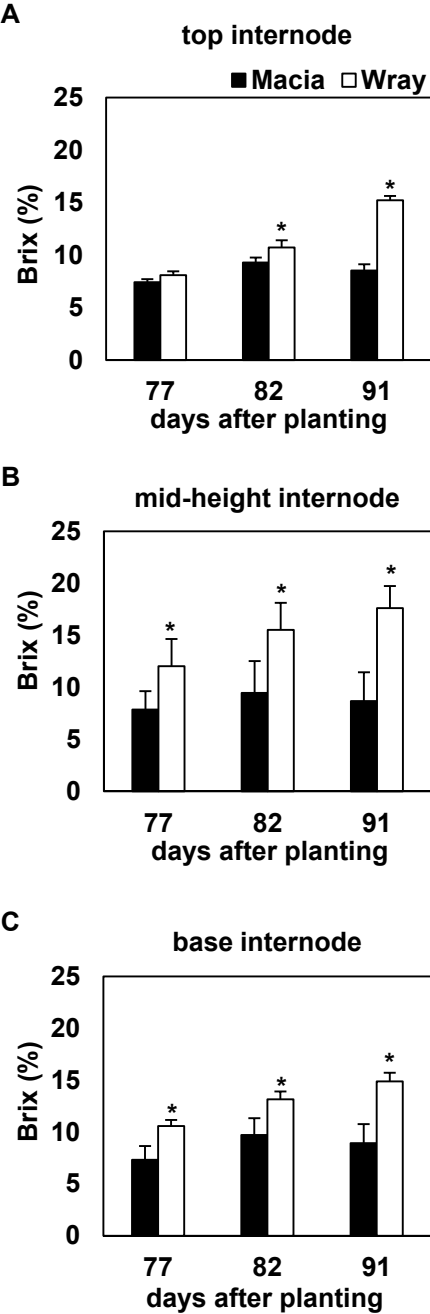

Supplement: Additional file 3: Figure S2. — Percent Brix of top (A), mid-height (B), and basal (C) internodes sampled on different days after planting. Values are means ± SE of N = 15, and an asterisk indicates significantly different means between the two lines at p ≤ 0.05. Macia = black boxes, and Wray = white boxes. (PDF 55 kb) [file 12870_2015_572_MOESM3_ESM.pdf]

Additional file 5: Figure S3

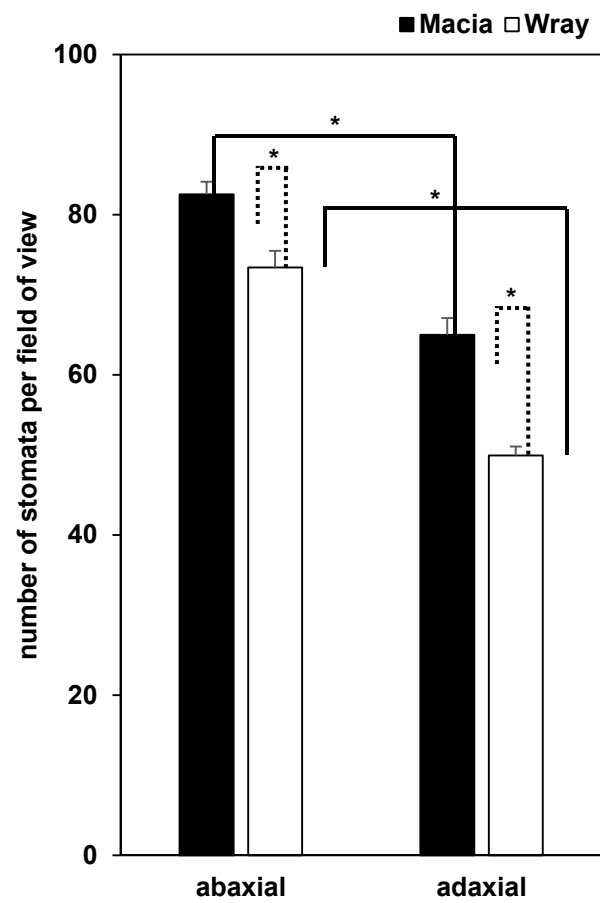

Supplement: Additional file 5: Figure S3. — Average number of stomata per field of view on the adaxial and abaxial leaf surfaces of field grown Macia and Wray plants. A total of N = 3 impressions were collected from each leaf surface of N = 5 plants per cultivar. Values are means ± SE, and an asterisk indicates significantly different means between the two lines for each surface or between opposite surfaces for each cultivar at p ≤ 0.05. Macia = black boxes, and Wray = white boxes. (PDF 51 kb) [file 12870_2015_572_MOESM5_ESM.pdf]

Additional file 6: Figure S4

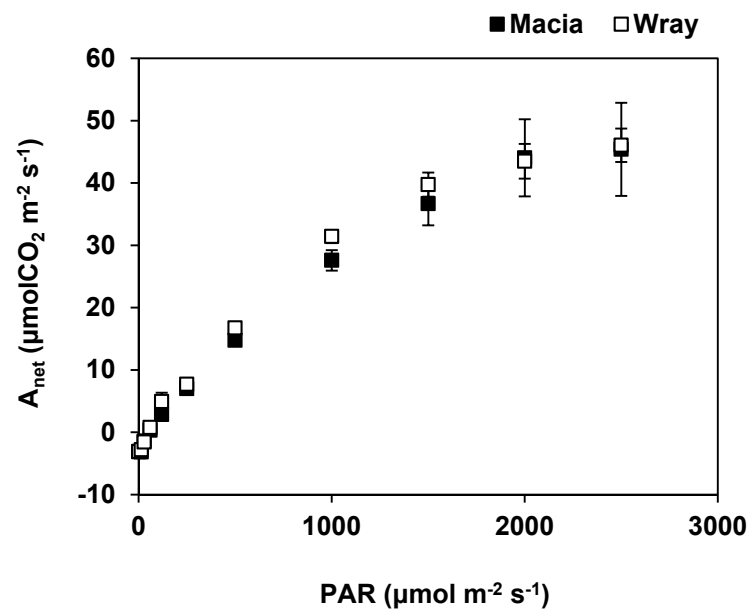

Supplement: Additional file 6: Figure S4. — Light response curves of Macia and Wray leaves from plants grown in the field, measured 64 days after planting. Net assimilation (A) is plotted on the y-axis, and photosynthetically active radiation (PAR) is plotted on the x-axis. Values are means ± SE of N = 5. Macia = black boxes, and Wray = white boxes. No statistical differences were detected between the two cultivars. (PDF 53 kb) [file 12870_2015_572_MOESM6_ESM.pdf]
