# Supplementary material for: Sucrose accumulation in sweet sorghum stems occurs by apoplasmic phloem unloading and does not involve differential Sucrose transporter expression
Source: BMC Plant Biol. 2015 Jul 30;15:186. doi: 10.1186/s12870-015-0572-8 (PMC4518677; doi:10.1186/s12870-015-0572-8)
Supplement: Additional file 4: Table S2. — Average Cq values ± SE obtained for each SbSUT gene in both leaf and stem tissues of Wray and Macia. (PDF 87 kb) [file 12870_2015_572_MOESM4_ESM.pdf]

Additional file 4: Table S2. Average Cq values ± SE for each *SbSUT* gene.

	Leaf		Stem	
	**Macia**	**Wray**	**Macia**	**Wray**
SbSUT1	26.9 ±0.17	27.2 ±0.15	27.9 ±0.13	28.2 ±0.17
SbSUT2	27.6 ±0.1	26.8 ±0.07	26.2 ±0.09	25.8 ±0.07
SbSUT3	31.6 ±0.06	31.7 ±0.09	31.8 ±0.13	33.1 ±0.13
SbSUT4	30.0 ±0.09	29.6 ±0.07	29.3 ±0.07	29.8 ±0.08
SbSUT5	31.4 ±0.08	31.5 ±0.14	31.5 ±0.15	33.0 ±0.19
SbSUT6	32.1 ±0.08	32.1 ±0.08	32.0 ±0.14	33.2 ±0.14
