# Supplementary material for: Sucrose accumulation in sweet sorghum stems occurs by apoplasmic phloem unloading and does not involve differential Sucrose transporter expression
Source: BMC Plant Biol. 2015 Jul 30;15:186. doi: 10.1186/s12870-015-0572-8 (PMC4518677; doi:10.1186/s12870-015-0572-8)
Supplement: Additional file 7: Table S3. — Chlorophyll fluorescence (Fv/Fm) measured on dark adapted leaves of Macia and Wray plants grown in the field. Measurements were taken 56 days after planting on N = 5. No statistical differences were detected between the two cultivars. (PDF 88 kb) [file 12870_2015_572_MOESM7_ESM.pdf]

Additional file 7: Table S3. Chlorophyll fluorescence (Fv/Fm) measured on dark adapted leaves of Macia and Wray field-grown plants.

**Cultivar**	**Fv/Fm**	**SE**	**p-value**
Macia	0.794	0.002	0.753
Wray	0.795	0.003	

Measurements were taken 56 days after planting on N = 5. No statistical differences were detected between the two cultivars.
