# Supplementary material for: Sucrose accumulation in sweet sorghum stems occurs by apoplasmic phloem unloading and does not involve differential Sucrose transporter expression
Source: BMC Plant Biol. 2015 Jul 30;15:186. doi: 10.1186/s12870-015-0572-8 (PMC4518677; doi:10.1186/s12870-015-0572-8)
Supplement: Additional file 8: Table S4. — List of PCR primer sets used in the qRT-PCR analyses. (PDF 80 kb) [file 12870_2015_572_MOESM8_ESM.pdf]

Additional file 8: Table S4. List of PCR primer sets used in the qRT-PCR analyses.

**Phytozome reference no.**	**Gene ID.**	**Primer name**	**Primer sequence (5' - 3')**	**Product size (bp)**
Sobic.001G488700	Sb01g045720	SbSUT1-3 F	ATGCCATCCGTTCTTCTCGT	124
Sobic.001G488700	Sb01g045720	SbSUT1-3 R	TCTGAGCATTGGTGCCCTTT	124
Sobic.008G193300	Sb08g023310	SbSUT2-2 F	TGCAATGGCTGCTAGTCGTGTT	145
Sobic.008G193300	Sb08g023310	SbSUT2-2 R	TGCATTTCCACCGCCGAACATT	145
Sobic.001G254000	Sb01g022430	SbSUT3-2 F	TTCCCGTTCCTGCAGACCAA	110
Sobic.001G254000	Sb01g022430	SbSUT3-2 R	AAGATCATGGTCACCACCGT	110
Sobic.004G353600	Sb04g038030	SbSUT4-2 F	ATGGAAACAACGTGTCGGCT	102
Sobic.004G353600	Sb04g038030	SbSUT4-2 R	AAACTGCTCCAGGTCCATCA	102
Sobic.004G190500	Sb04g023860	SbSUT5-MF (Milne et al., 2013)	CCCGTAGTGTTGCGGAGTC	119
Sobic.004G190500	Sb04g023860	SbSUT5-MR (Milne et al., 2013)	CCAATGGATCGGAAAATAAAG	119
Sobic.007G214500	Sb07g028120	SbSUT6-1 F	CTCCTCTTCTGCTCCGTCGC	108
Sobic.007G214500	Sb07g028120	SbSUT6-1 R	GAACACCATGAGGTTGCTGA	108
pSP64poly(A)-luc	n/a	Luc-F	CCAGGGATTTCAGTGGATGT	183
pSP64poly(A)-luc	n/a	Luc-R	AATCTGACGCAGGCAGTTCT	183
